# Cardiac overload resolved by resection of a large plexiform neurofibroma on both the buttocks and upper posterior thighs in a patient with neurofibromatosis type I: a case report

**DOI:** 10.1186/s12893-020-00761-4

**Published:** 2020-05-18

**Authors:** Taro Mikami, Yuki Honma-Koretsune, Yui Tsunoda, Shintaro Kagimoto, Yuichiro Yabuki, Jiro Maegawa, Miki Terauchi, Shintaro Nawata, Hiroyuki Kamide, Yoshinobu Ishiwata, Tabito Kino, Teruyasu Sugano

**Affiliations:** 1 Department of Plastic and Reconstructive Surgery, Chigasaki Municipal Hospital, 253-0042, Honson 5-15-1, Chigasaki, Kanagawa Japan; 2 Department of Plastic and Reconstructive Surgery, Yokohama City University, School of Medicine, Yokohama, Japan; 3 Department of Radiology, Yokohama City University, School of Medicine, Yokohama, Japan; 4 Department of Radiology, Yokohama City University Medical Center, Yokohama, Japan; 5 Department of Medical Science and Cardiorenal Medicine, Yokohama City University, School of Medicine, Yokohama, Japan

**Keywords:** Body weight, Cardiac overload, Echocardiography, Embolism, Plexiform neurofibroma, Neurofibromatosis, Malnutrition, Skin transplantation, Tricuspid valve insufficiency, Quality of life

## Abstract

**Background:**

A large plexiform neurofibroma in patients with neurofibromatosis type I can be life threatening due to possible massive bleeding within the lesion. Although the literature includes many reports that describe the plexiform neurofibroma size and weight or strategies for their surgical treatment, few have discussed their possible physical or mental benefits, such as reducing cardiac stress. In addition, resection of these large tumors can result in impaired wound healing, partly due to massive blood loss during surgery.

**Case presentation:**

A 24-year-old man was diagnosed with neurofibromatosis type I and burdened with a large plexiform neurofibroma on the buttocks and upper posterior thighs. The patient was 159 cm in height and 70.0 kg in weight at the first visit. Cardiac overload was indicated by an echocardiography before surgery. His cardiac output was 5.2 L/min with mild tricuspid regurgitation. After embolism of the arteries feeding the tumor, the patient underwent surgery to remove the neurofibroma, followed by skin grafting. Follow-up echocardiography, performed 6 months after the final surgery, indicated a decreased cardiac output (3.6 L/min) with improvement of tricuspid regurgitation. Because the blood loss during the first surgery was over 3.8 L, malnutrition with albuminemia was induced and half of the skin graft did not attach. Nutritional support to improve the albuminemia produced better results following a second surgery to repair the skin wound.

**Conclusion:**

Cardiac overload may be latent in patients with neurofibromatosis type I with large plexiform neurofibromas. As in pregnancy, the body may compensate for this burden. In these patients, one stage total excision may improve quality of life and reduce cardiac overload. In addition, nutritional support is likely needed following a major surgery that results in either an extensive skin wound or excessive blood loss during treatment.

## Background

Plexiform neurofibromas are sometimes observed in patients with neurofibromatosis type I [1]. Often, plexiform neurofibromas are highly vascularized, which can place the patient at an increased risk of intratumoral hemorrhage following blunt trauma [2]. The literature includes case reports of adults who have undergone total resection, even under an emergency situation, and recommends total over partial resection in pediatric patients undergoing elective surgery [3, 4]. Embolization of arteries feeding the tumor has been shown to effectively reduce blood loss during surgery [3, 5–7]. Although several case reports described surgeries where the weight of the resected tumor was over 10 kg, few articles have reported a change in cardiac function after surgery compared to before, and most have only commented that the clinical course following surgery was uneventful.

A patient with plexiform neurofibroma due to neurofibromatosis type I (NF-1) underwent surgical treatment at our institute. His body weight was approximately 80 kg before surgery and the excised tumor weighed almost 30 kg. In this report, we describe his clinical course including pre- and post-surgery echocardiograms.

## Case presentation

A 24-year-old man presented to our hospital with a complaint of instability during exercise and difficulty in defecation. When the patient was 14 years old, classmates had pointed out his enlarged buttocks. He consulted a doctor at a nearby hospital and was diagnosed with neurofibromatosis type I based on clinical findings on physical examination. When he was 20 years old, he underwent partial resection of the lesion because the tumor interfered with defecation. During surgery, the patient suffered blood loss of up to 800 ml within an hour. Since the lesion continued to grow after this partial resection, a second surgery was planned. Because the previous surgery had incurred a large blood loss, the patient was treated by a multidisciplinary team at the university hospital.

**Fig. 1:**
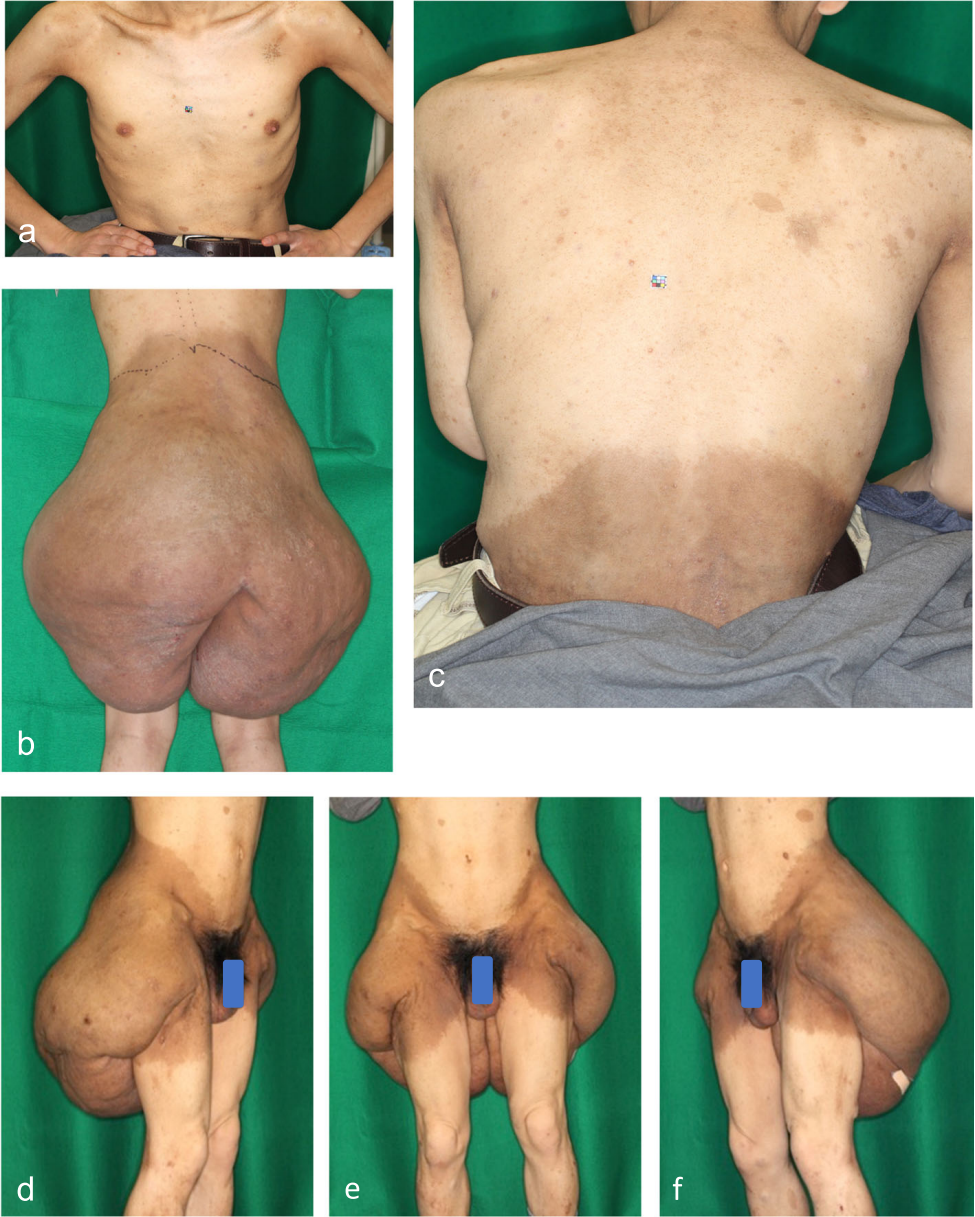
Local findings of the patients before surgery. **a**. Local status of the chest, upper abdomen, and upper arm. Small size tumors were observed on the left upper arm and the anterior chest. These are thought to be neurofibromas. **b**. Findings of the lower chest, buttocks, and thigh of posterior side. Large tumor with dark brown color that is almost symmetrical is observed. **c**. Local findings of the patient’s upper back. There is no plexiform neurofibroma in the upper back of the patient. Some small size café-au-lait spots are observed around the scapula. **d**. The patient‘s right anterior oblique finding. The tumor is drooped on the lateral and posterior side of the right thigh. **e**. The anterior finding of the pelvis and thighs of the patient. Large size café-au-lait spots are noticed in the bilateral anterior thigh. **f**. The patient’s left anterior oblique finding. The tumor is drooped on the lateral and posterior side of the left thigh

At his first visit the patient was 159 cm in height and 70.0 kg in weight without an obvious osseous lesion. A large tumor was located on the patient’s bilateral buttocks and posterior-upper thighs. A few large café-au-lait spots were observed, as were small skin tumors on his trunk and limbs, which were diagnosed as neurofibromas. No intracranial lesion was identified (Fig. 1).

Otherwise, the patient’s medical history was unremarkable with no family history of type I neurofibromatosis. He had worked as a transporter until the large tumor on his buttocks became a burden. He regularly exercised and ran to maintain muscle strength.

Large, bilateral, and almost symmetric drooping tumors were observed on the buttocks. The surface of the tumor was smooth, hairless, and shiny, and the margin of the tumor was relatively unclear. Both the gluteal cleft and the gluteal sulcus were indistinct (Fig. 1). Although the large lesion interfered with daily life, the patient was not aware of exertion dyspnea or palpitations. Ultrasound cardiography was performed one month before admission. The patient’s cardiac output was relatively high (5.2 L/min), and tricuspid regurgitation was observed.

### Imaging

**Fig. 2:**
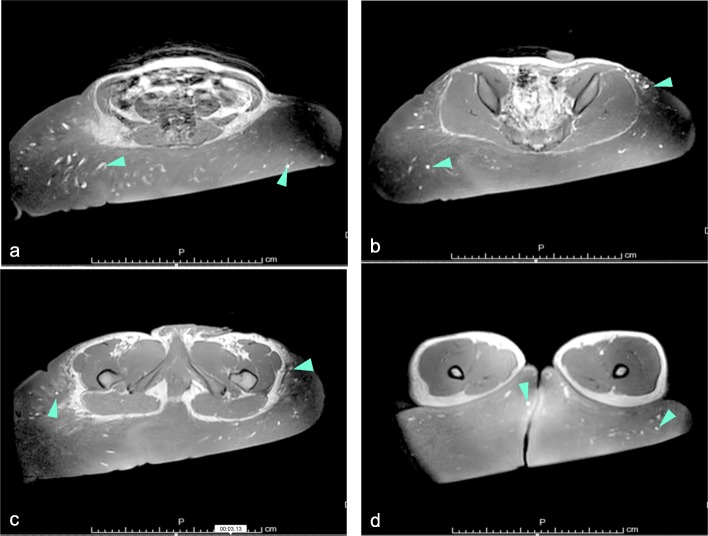
T2 weighted images (T2WI) of the patient’s lesion before surgery. **a** .Superior side of the lumbar area of the lesion. **b**. Buttocks and sacral area of the lesion. **c**. Upper thigh area of the lesion. **d**. Lower thigh area of the lesion. The plexiform neurofibroma shows relatively high intensity in T2WI of MRI. There are many high intensity signal lesions in the images (green arrow head). These are considered to be dilated veins or arteries


Magnetic resonance imaging (MRI), which was performed at the previous hospital, showed a large tumor on the muscle layer in a bilateral area from the lumbar to the posterior thigh (Fig. 2, supplemental data 1, 2). The lesion showed almost homogeneous isointensity relative to the muscle on T1-weighted images (T1WI) and showed a slightly higher intensity signal relative to the muscle in T2-weighted images (T2WI). Many luminal organs, with diameters up to 1 cm, were observed on the MR images. These were identified as blood vessels.

**Additional file 1: Supplemental data 1.** Video of T1-weighted images of the patient before surgery.


**Additional file 2: Supplemental data 2.** Video of T2-weighted images of the patient before surgery.

A contrast-enhanced computed tomography (CT) scan was performed one month after the first visit. The tumor appeared as a homogeneous low-density area in the early phase including enhanced vessels (supplemental data 3, 4, 5). Another thin, low-density area was observed between the muscles of the erector spinae, bilateral gluteus maximus, posterior side of the thighs, and the large tumor in both early phase and late phase. This indicated the presence of loose tissue between the large lesion and the muscle layer (supplemental data 3, 4, 5).


**Additional file 3: Supplemental data 3.** The video shows early phase images of the dynamic enhanced CT. High-density linear lesions in the tumor are regarded as arteries


**Additional file 4: Supplemental data 4.** The video shows late phase images of the dynamic enhanced CT. High-density linear lesions in the tumor are regarded as veins including sinus vessels. In addition, the plexiform neurofibroma is enhanced homogeneously, comparing with early phase images.

### Surgical treatment

**Fig. 3:**
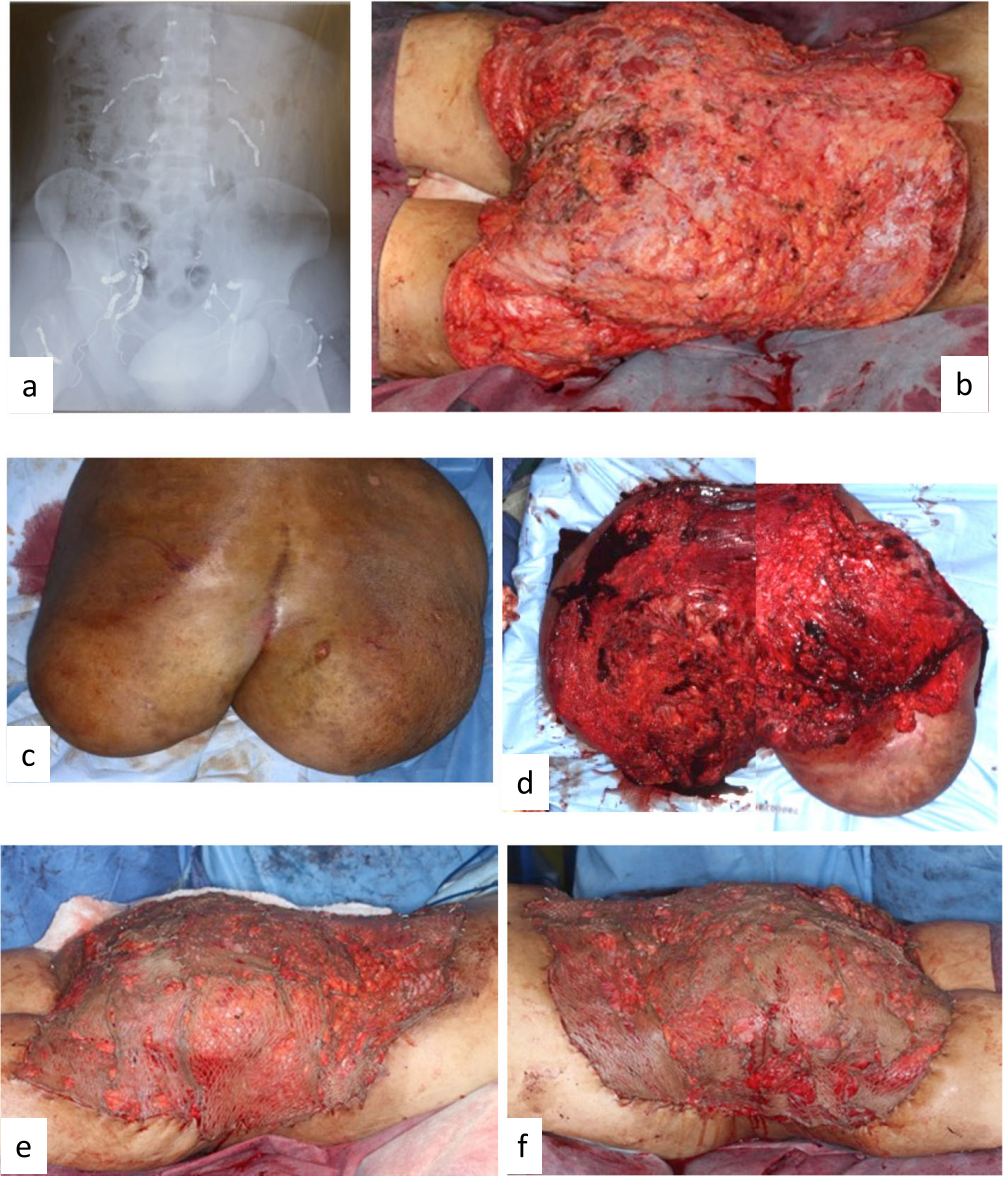
Preoperative and intra operative findings of the patient. **a**. The x-ray of the abdomen and the pelvis taken after the intervention radiology. Many coils are presented in the picture as white-line shadows. **b**. The raw surface of the lower back, bilateral buttock, and the upper posterior thigh. Almost all of the surface is the subfascial plane, just above the muscles. **c**. The outer surface of the resected tumor. A linear post-operative scar is observed on the center of the surface. **d**. The inner surface of the resected tumor. Two pictures are bound to one picture because the tumor was too large to take photo as one piece. **e**. The right-side aspect just after skin grafting. The skin graft was taken from the resected tumor by electric dermatome. **f**. The left side aspect just after skin grafting. The skin graft had been processed with mesh dermatome as 1 to 3 mesh skin graft

Based on the literature, we planned to use interventional radiology (IVR) to embolize the arteries feeding the plexiform neurofibroma followed soon after by a total excision of the tumor. Two weeks before the day of surgery, the patient was admitted to Yokohama City University Hospital, where he underwent embolization on the next, fourth, and eighth days. In the first procedure, the 2^nd^ lumbar arteries on each side, the 3^rd^ lumbar artery on the left side, and the 4^th^ lumbar artery on the right side were embolized. During the second procedure, all other lumbar arteries underwent IVR embolization. At the same time, the bilateral superior gluteal arteries, right inferior gluteal artery, left superficial circumflex iliac artery, and left lateral circumflex femoral artery were embolized. In the last procedure, the right lateral circumflex femoral artery, a branch of the right superior gluteal artery, and a branch of the left inferior gluteal artery were embolized. Only the right superficial circumflex iliac artery was identified as not requiring embolization. Platinum micro coils were used in all embolization procedures (Fig. 3).

The first excision surgery was performed two days after the last IVR treatment. The incision line was located approximately 1 cm to the lateral side of the tumor margin as based on CT and MRI. Under general anesthesia, in the lateral position of both sides, dissection with electric scalpel was performed just under the deep fascia (Fig. 3) [8]. The boundary between the tumor and the muscle was clear in almost all areas, and only a little arterial bleeding was observed. However, venous bleeding was obvious, especially from the surface of the dissected tumor, and hemostasis with ligation was needed. The lesion around the anus was the last section to be treated. Surgeons from the Department of Gastroenterological Surgery treated this area, and the tumor was totally removed en bloc. Total operation time was 14 hours and 15 minutes with four position changes, and blood loss was 3862 grams. The patient was transfused with 20 units of packed red blood cells, 20 units of fresh frozen plasma (FFP), and 2 units of autologous blood prepared before surgery. After removal of the tumor, a skin graft was taken from the surface of the excised lesion. The graft was applied as a 3:1 mesh skin graft using a modified Alabama method (Fig. 3). The patient was sent to the intensive care unit (ICU) after extubation, observed for 10 hours, and then moved to the general ward.

The patient’s clinical course was mostly uneventful. Although only half of the skin graft had attached, there was no sign of local infection. The nutrition support team intervened two weeks after surgery because the patient’s hypoalbuminemia (2.0 to 2.5 g/dl), which was thought to be caused by blood loss during surgery, had not improved. Once the albumin levels reached 3.3 g/dl, the next operation was planned. The second surgery was performed 34 days after the first surgery. An approximately 400 cm^2^ of split thickness skin graft was removed from the back. Part of the graft was applied as a 3:1 mesh skin graft while the remaining part was grafted as a patch skin graft (supplemental data 6). The patient was discharged following epithelialization of the donor site.

### Clinical course after discharge

**Fig. 4:**
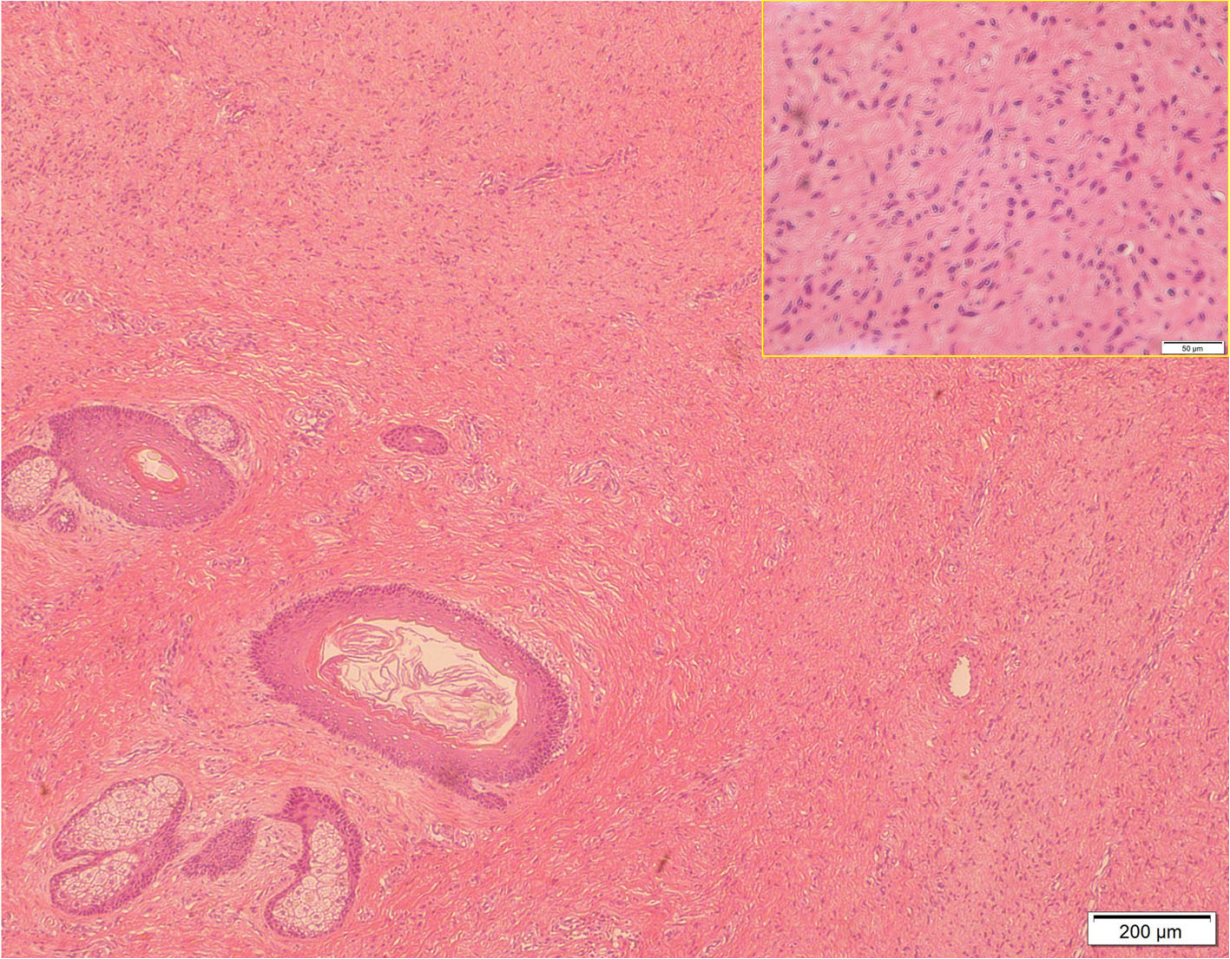
Histopathological findings of the resected tumor. The picture shows a specimen of the resected lesion. Epithelial inclusion cysts and sebaceous sweat glands surrounded by fibroplasia are observed in this slice. The inlet shows a part of this slice in a high-power field. Many of the cells have wavy spindle nuclei while few mitoses are observed. No findings indicating malignancy were noticed in any of the slices, including this slice. The scale bars in the main part and inlet show 200 μm and 50 μm, respectively

**Fig. 5:**
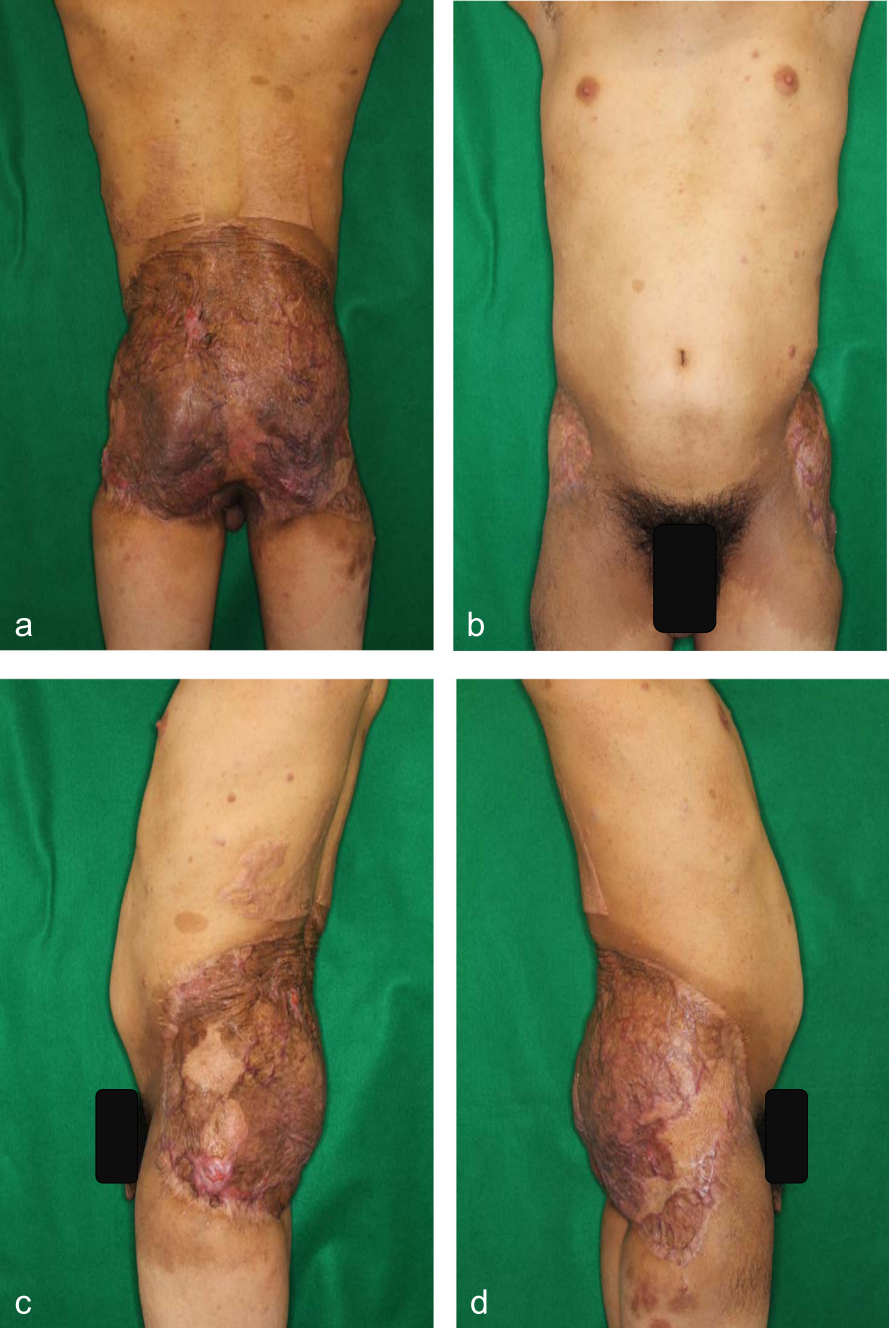
Local findings of the patient one year after surgery. **a**. Dorsal side. The skin graft has been matured with only few hypertrophic scars. The scars in the lower back due to skin graft harvesting are not so obvious. **b**. Ventral side. Lateral margins of the skin graft can be observed in this view. There is no evidence of scar contracture. **c**. and **d**. Findings of the bilateral sides. The sheet skin grafts by the second surgery are remarkable. The wide scars in the upper and lower margins of the lateral areas of the skin graft are not so hypertrophic


Although erosion occurred in a few areas of the skin graft, conservative therapy with an ointment (Actosin® Ointment, Maruho Co, Ltd, Osaka, Japan) recovered epithelialization. The patient noticed improvement in daily activities due to his weight loss. A histopathological examination of the tumor did not indicate malignancy (Fig. 4). Eighteen months after the primary surgery, clinical findings revealed no obvious recurrence, although follow-up CT images indicated a small tumor in the right back and the left buttock (Fig. 5, supplemental data 7).

**Additional file 7: Supplemental data 7.** The video shows the status 1.5 year after surgical treatment. Almost all of the tumor was resected except for the right upper lumbar area.

An echocardiogram was performed 8 months after the resection surgery. A significant decrease in cardiac output from 5.2 L/min to 3.6 L/min was observed and tricuspid regurgitation had improved. In addition, the size of the left atrium and ventricle and the pericardial effusion had decreased (Table 1).

**Table 1:** Summary of echocardiographic data before and after surgical treatments

		Pre op	Post op
Height	cm	159.0	159.0
Body Weight	kg	75.2	56.8
Body Surface Area	m^2^	1.8	1.6
IVSd	mm	8.1	9.0
IVSs	mm	10.6	12.1
AoD	mm	26.4	28.5
LVDd	mm	48.6	43.2
LVDs	mm	29.8	27.7
LAD	mm	37.8	32.6
LVPWd	mm	8.3	8.2
LVPWs	mm	12.1	12.0
Ejection Fraction	%(2D)	69	66
Cardiac Output	L/min(2D)	5.2	3.6
Cardiac Index	L/min/ m^2^ (2D)	2.9	2.3
A valve		N.P.	N.P.
M valve		MR; trivial	MR; trivial
T valve		TR; mild	TR; trivial
P valve		PR; trivial,End-diastolic PG = 2 mmHgEnd-systolic PG = 6 mmHg	PR; trivial, End-diastolic PG = 3 mmHgEnd-systolic PG = 9 mmHg

The data before surgical treatments were taken one month before surgery, and post operation data were obtained 8 months after the resection surgery. These data indicate latent cardiac overload before surgery that has been improved by total resection of the plexiform neurofibroma. Pre op: before operation, Post op: after surgical treatments, IVSd: Interventricular septal end diastolic dimension, IVSs: Interventricular septal end systolic dimension, AoD: Aortic root diameter, LVDd: Left ventricular end diastolic dimension, LVDs: Left ventricular end systolic dimension, LAD: Left atrial dimension, LVPWd: Left ventricular end diastolic posterior wall dimension, LVPWs: Left ventricular end systolic posterior wall dimension, A valve: Aortic valve, M valve: Mitral valve, T valve: Tricuspid valve, P valve: Pulmonary valve, MR: Mitral regurgitation, TR: Tricuspid regurgitation, PR: Pulmonary regurgitation

## Discussion and conclusions

This report provides two new findings. First, the patient appeared to show less cardiac stress following the resection surgery. Secondly, malnutrition due to excessive bleeding, such as that observed during surgery for resection of a large plexiform neurofibroma, may result in delayed wound healing and lead to skin graft failure or necrosis of skin flaps.

Plexiform neurofibroma is a characteristic of neurofibromatosis type I. Neurofibroma growth, depending on its location, can interfere with daily living activities. In some instances, massive bleeding within the tumor can lead to hemorrhagic shock, and surgical treatment is necessary. There are two possible approaches to excision of the tumor, one stage total excision and serial excision.

The literature indicates that one stage total resection is recommended in younger patients (10 to 15 years of age) because the recurrence rate with this approach is significantly lower than with partial resection [4]. Though our patient was an adult, we performed one stage total resection once we had been informed that a previous surgery involving a partial resection had induced massive bleeding from the cutting plane of the tumor.

MRI and CT imaging indicated that the tumor contained many dilated vascular beds, so we decided to use IVR to embolize the arteries feeding the plexiform neurofibroma. In addition, the patient`s heart may have been overloaded while circulating the larger volume of blood

We calculated that the circulating blood volume decreased from 5.78 L to 4.37 L following surgery, decreasing the cardiac output. Tricuspid regurgitation, left atrial enlargement, and left ventricular enlargement, which had been observed before the operation, all improved post-surgery, perhaps because the load on the right ventricles had decreased. On the other hand, the cardiac index as well as the cardiac output was within the normal limit. In addition, the ability of the left ventricle to expand had not changed. The increased cardiac load from the large plexiform neurofibroma of this patient was considered similar to that in pregnancy.

Few articles have discussed the effects that resecting giant neurofibromas might have on cardiac load, perhaps because subjective symptoms such as exertion dyspnea or palpitation that result from cardiac overload are not generally reported in patients with NF-1 with large plexiform neurofibroma. In fact, our patient had not felt dyspnea or palpitation due to cardiac overload while training via long-distance running or jogging before surgery. According to the patient’s echocardiograms, however, a large plexiform neurofibroma may indeed lead to cardiac overload even when it asymptomatic. We suggest therefore that removal of a large plexiform neurofibroma can be beneficial by reducing cardiac overload, as well as for preventing malignant change within the tumor, avoiding accidental massive bleeding in the tumor that can cause hemorrhagic shock, and improving a patient’s quality of life [9].

Grafting of skin taken from the resected tumor to the raw surface is thought to be an effective option for reconstructive surgery in this type of patient. However, the massive bleeding associated with removal of a large plexiform neurofibroma may produce malnutrition that leads to partial necrosis of the skin graft, although the recurrence rate may be reduced comparing with covering by skin flap [10]. In this case, the raw surface after resection of the large lesion was estimated as 10% of the total body surface area. As the blood loss during the first surgery was estimated at 65%, a certain level of serum protein including albumin was lost. The patient’s position after the first surgery might have been a factor for failure in skin grafting. In fact, one article has reported the importance of remaining in a prone position for four days after surgery of a lesion on the back to promote wound healing [8]. Nevertheless, intervention by the nutrition support team improved both malnutrition and the condition of the wound, suggesting that malnutrition was the main cause of skin graft failure. There are a few reports on the importance of nutrition control after resection of large plexiform neurofibromas [10, 11]. Including nutritional support early after excision surgery may improve the success of skin grafting to the raw surface and avoid a delay in wound healing.

## Conclusion

In conclusion, cardiac overload may be latent in patients with large size plexiform neurofibromas, and patients may compensate for increased cardiac output, as observed in pregnancy. Therefore, one stage total excision is presumed to be beneficial not only for improved quality of life but also for reduction in any cardiac overload. However, additional patient data are needed to confirm this hypothesis. Skin grafting from the resected lesion may help prevent tumor recurrence following a one-stage total resection. Nutritional support is presumed necessary when surgery produces an extensive wound or when excessive blood loss has occurred during surgery.

## Supplementary information

**Additional file 5: Supplemental data 5.** The video shows reconstructed sagittal images of early phase images of the dynamic enhanced CT. Arteries in the tumor seem to be arising from the deep layer of the muscle and some of them seem to be coiling in the tumor. Maximum size of the arteries is up to 1 cm.

**Additional file 6: Supplemental data 6.** Local findings in the second surgery. a. Right posterior oblique view of the patient before the second surgery. About 80% of the tumor-resected area was covered by the first surgery. The raw surface is red-colored without sign of infection. Main area of the raw surface seems to be located in the weight-bearing area in the right lateral decubitus position. b. Left posterior oblique view of the patient before the second surgery. About 70% of the tumor-resected area was covered by the first surgery. The main area of the raw surface is considered to be located in the weight-bearing area in the left lateral decubitus position. c. Posterior view of the patient before the second surgery. The raw surface is located on the center of the sacral region. A part of the native skin around the anus is observed in this picture. d. Right posterior oblique view of the patient just after the second surgery. The split thickness skin grafts were dressed by modified Alabama method with surgical sponges, while the patch skin graft was covered by Aquacel ® Ag burn. The donor site was covered by sheets of gauze in this picture.

## Data Availability

The data and supplementary data are available from the corresponding author upon a reasonable request.

## Abbreviations

NF-1: neurofibromatosis type I
MRI: magnetic resonance imaging
CT: computed tomography
IVR: interventional radiology
FFP: fresh frozen plasma
ICU: intensive care unit
