# Correction: Cardiac overload resolved by resection of a large plexiform neurofibroma on both the buttocks and upper posterior thighs in a patient with neurofibromatosis type I: a case report

**DOI:** 10.1186/s12893-024-02591-0

**Published:** 2024-10-26

**Authors:** Taro Mikami, Yuki Honma-Koretsune, Yui Tsunoda, Shintaro Kagimoto, Yuichiro Yabuki, Jiro Maegawa, Miki Terauchi, Shintaro Nawata, Hiroyuki Kamide, Yoshinobu Ishiwata, Tabito Kino, Teruyasu Sugano

**Affiliations:** 1 Department of Plastic and Reconstructive Surgery, Chigasaki Municipal Hospital, 253-0042, Honson 5-15-1, Chigasaki, Kanagawa Japan; 2 Department of Plastic and Reconstructive Surgery, Yokohama City University, School of Medicine, Yokohama, Japan; 3 Department of Radiology, Yokohama City University, School of Medicine, Yokohama, Japan; 4 Department of Radiology, Yokohama City University Medical Center, Yokohama, Japan; 5 Department of Medical Science and Cardiorenal Medicine, Yokohama City University, School of Medicine, Yokohama, Japan

**Correction: BMC Surgery (2020) 20:106**

10.1186/s12893-020-00761-4

Following publication of the original article [1], the author’s name Miki Terauchi was incorrectly written as Takashi Terauchi.

The original article has been corrected.
