# Supplementary figures and images for: Cardiac overload resolved by resection of a large plexiform neurofibroma on both the buttocks and upper posterior thighs in a patient with neurofibromatosis type I: a case report
Source: BMC Surg. 2020 May 18;20:106. doi: 10.1186/s12893-020-00761-4 (PMC7236506; doi:10.1186/s12893-020-00761-4)

## Slide 1
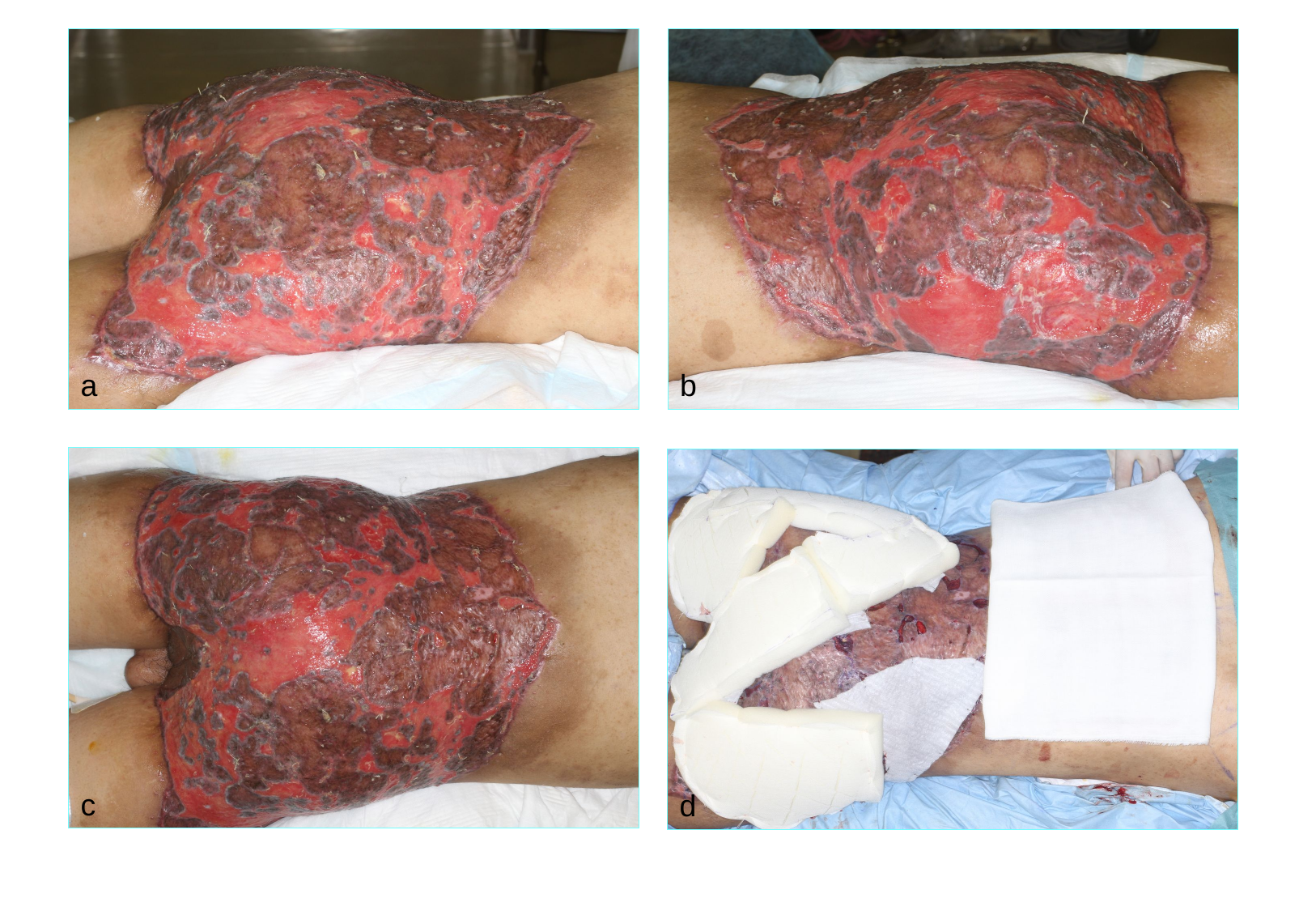

a
b
c
d

Supplement: Supplementary file 7 — Additional file 6: Supplemental data 6. Local findings in the second surgery. a. Right posterior oblique view of the patient before the second surgery. About 80% of the tumor-resected area was covered by the first surgery. The raw surface is red-colored without sign of infection. Main area of the raw surface seems to be located in the weight-bearing area in the right lateral decubitus position. b. Left posterior oblique view of the patient before the second surgery. About 70% of the tumor-resected area was covered by the first surgery. The main area of the raw surface is considered to be located in the weight-bearing area in the left lateral decubitus position. c. Posterior view of the patient before the second surgery. The raw surface is located on the center of the sacral region. A part of the native skin around the anus is observed in this picture. d. Right posterior oblique view of the patient just after the second surgery. The split thickness skin grafts were dressed by modified Alabama method with surgical sponges, while the patch skin graft was covered by Aquacel ® Ag burn. The donor site was covered by sheets of gauze in this picture. [file 12893_2020_761_MOESM6_ESM.pptx]
